# Supplementary material for: Assessing changes in frailty status in an elderly population: Analysis of results from the SFGE tool
Source: PLoS One. 2025 Jul 14;20(7):e0327227. doi: 10.1371/journal.pone.0327227 (PMC12258554; doi:10.1371/journal.pone.0327227)
Supplement: Attachment — Submitted filename: Response to Review of Manuscript.docx [file pone.0327227.s003.docx]

**Response to Review of Manuscript**

Dear Academic Editor

We thank you and the reviewers for your insightful comments and constructive feedback on our manuscript, **“**Assessing changes in frailty status in an elderly population: Analysis of results from the SFGE tool”. We have carefully considered each point raised and have made significant revisions to the manuscript to address these concerns. Below, we provide a point-by-point response to the reviewers' comments, outlining the changes made and providing clarifications.

**Response to Reviewer Comments:**

**Point 1: Ambiguous Definition and Lack of Description of the "Individualized Care Plan"**

We acknowledge the reviewer's concern regarding the definition and description of the "Individualized Care Plan" (ICP). We recognize that the lack of detail in the original manuscript limited the interpretability of our findings. To address this:

- We have significantly expanded Section "Methods"to provide a detailed description of the ICP development process, its key components, and the multidisciplinary team involved.
- Specifically, we have included information on how the ICPs were tailored to address patient-specific needs identified during the comprehensive geriatric assessment, including the key determinants of social frailty. We now explicitly state whether and how social isolation, lack of social support, limited access to resources, and other social factors were addressed within the ICPs.

**Point 2: Complementary Analysis Exploring Specific Items Driving Improvements in Frailty**

We appreciate the reviewer's suggestion for a complementary analysis. While our primary analysis focused on the overall change in frailty scores, we agree that exploring specific items driving these changes would provide valuable insights.

- We have now included a supplementary analysis (presented in Supplementary Table) that examines the changes in individual items of the SFGE.

**Point 3: Incorporation of the "Sunfrail+" Tool**

We understand the reviewer's query regarding the incorporation of the "Sunfrail+" tool.

- We have clarified in the Methods section how the "Sunfrail+" tool was utilized in our study. We have explicitly stated the specific components of the "Sunfrail+" tool that were employed

**Point 4: Convenience Sampling and Limitations of SFGE**

We acknowledge the limitations associated with the use of convenience sampling and the nature of the SFGE.

- In the Limitations section of the Discussion we have included a thorough discussion on the potential biases introduced by convenience sampling and how this might affect the generalizability of our findings. We have also acknowledged the characteristics of our study population and the potential for selection bias.

**Point 5: Comparison of Multidimensional Versus Physical Frailty Models**

We appreciate the reviewer's suggestion to expand the discussion by comparing the utility of multidimensional versus physical frailty models and for providing the relevant citation.

- We have now incorporated a dedicated paragraph in the Discussion section that discusses the findings of Testa et al. (2020) and compares the utility of multidimensional frailty assessments (like the SFGE) with purely physical frailty models in the context of our study population and outcomes.

**Point 6: Language Revision and Graphical Presentation**

We thank the reviewer for pointing out the grammatical errors and suggesting improvements to the graphical presentation.

- We have carefully reviewed the entire manuscript and have made thorough language revisions to correct all identified grammatical errors and improve the overall quality of English.

We believe that these revisions have significantly strengthened our manuscript and addressed the concerns raised by the academic editor and reviewer. We are confident that the revised manuscript provides a clearer and more comprehensive understanding of our study and its findings.

Thank you once again for your valuable feedback. We look forward to your positive consideration of our revised manuscript.

Sincerely

Dott. Edoardo Carnevale

Department of Biomedicine and Prevention – University of Rome Tor Vergata

Edoardocarnevale1@gmail.com
